# Investigating the relationship between the occlusal plane and the tragus-ala line in patients with different jaw skeletons

**DOI:** 10.4317/jced.63151

**Published:** 2025-10-01

**Authors:** Hadi Ranjzad, Farzaneh Ostovarrad, Elnaz Saidi, Mahyar Eftekhar, Zahra Ghorbani

**Affiliations:** 1 Assistant Professor, Department of Prosthodontics, School of Dentistry, Gilan University of MedicalSciences, Rasht, Iran; 2 Associate Professor, Dental Science Research Center, Department of Oral and Maxillofacial Radiology, School of Dentistry, Gilan University of Medical Sciences, Rasht, Iran; 3 Dentist, Anzali Faculty of Dentistry, International Branch, Guilan University of Medical Sciences, Rasht, Iran; 4 Department of Prosthodontics , School of Dentistry, Dental Sciences Research Center , Gilan University of Medical Sciences , Rasht, Iran

## Abstract

**Background:**

This study investigated the relationship between the occlusal plane and the ala-tragus lines (ATL) for restoring the upper and lower jaws and analyzed the ATL in different jaw classifications.

**Material and Methods:**

This analytical cross-sectional study included lateral cephalometric radiographs of 172 patients with teeth in a normal position. The three ATLs and the occlusal plane were delineated, and jaw classification was determined using Waits and Steiner analysis.

**Results:**

For jaw classes 1 and 2, the parallelism between the lower ATL and the occlusal plane was not statistically significant. However, for class 3 patients, significant parallelism was observed between the lower ATL and the occlusal plane, regardless of the maxillary classification. In the gender-based analysis, the parallelism between the occlusal plane and the lower ATL was significant for women. In the age-based analysis, the parallelism was significant in the age group of 15–21 years.

**Conclusions:**

These findings suggest that the lower ATL can be a reliable reference for determining the occlusal plane. The study also highlights that age and gender are important factors influencing the determination of the occlusal plane using the ATL.

** Key words:**Occlusal plan, ala-tragus line, cephalometric radiograph.

## Introduction

The current glossary of dental prosthetic terms defines the occlusal plane as “the surface created by the incisal and occlusal surfaces of the teeth.” However, in practice, the occlusal plane is rarely a flat surface and instead represents the average curvature of these surfaces [1]. Determining and adjusting the occlusal plane is an essential clinical procedure in the complete reconstruction of the upper and lower jaws [1]. The proper orientation of the occlusal plane is critical for achieving aesthetic and functional satisfaction [2]. An incorrect occlusal plane orientation compromises the harmony of the joint complex within the oral and facial structures [3]. When the occlusal plane in fixed or removable prostheses is improperly oriented, it can disrupt the interaction between the tongue and the buccinator muscle, leading to food accumulation in the sulcus, accidental biting of the cheek or tongue, and accelerated resorption of the remaining alveolar ridge due to improper force distribution on the ridge [4]. In cases where the occlusal plane or teeth are defective, using anatomical markers to adjust the ideal occlusal plane becomes crucial. Several anatomical landmarks have been proposed to determine the occlusal plane clinically. These include the upper lip, lateral margins of the tongue, the ATL, and the incisive papilla-hamular notch plane [5]. The ATL is drawn from the nasal ala’s lower border to a point on the tragus of the ear, typically the tip of the tragus. Ideally, this line is considered parallel to the occlusal plane. The ATL is one of the most frequently used extra oral landmarks for determining the posterior occlusal plane in edentulous patients. However, its use remains controversial, primarily due to disagreements about the exact reference point on the tragus (whether it should be the superior, middle, or inferior point) to establish the tragus contour [6]. Given the importance of these issues, this study aims to investigate the relationship between the occlusal plane and the ATL as a guide for reconstructing the occlusal plane in patients who are candidates for complete upper and lower jaw reconstruction across different jaw classifications.

## Material and Methods

This study examined 172 patients (49 males and 123 females, aged 15-45 years) who were prescribed lateral cephalometric radiography. This analytical cross-sectional study excluded patients with a history of orthodontic treatment, prosthodontic procedures, facial surgeries (e.g., rhinoplasty), abnormal teeth positioning, lack of occlusion on both sides of the maxilla, or defects in the normal anatomy of the facial structures.

To establish a reference point for the tragus (middle tragus), adhesive tape was attached to a 2 mm mammography skin marker to the center of the patient’s tragus. The vertical height of the tragus was measured with a digital caliper, and the measurements were divided across the upper, middle, and lower tragus points to define the three ATLs. Lateral cephalometric images drew these ATLs corresponding to the nasal ala and the occlusal plane. The following lines were defined:

• Ala tragus line superior (ATLs): A line extending from the nasal ala’s lower border to the tragus’s upper border.

• Ala tragus line middle (ATLm): A line extending from the lower border of the nasal ala to the middle point (tip) of the tragus.

• Ala tragus line inferior (ATLi): A line extending from the lower margin of the alatragus to the lower border of the tragus of the ear.

Jaw classification was determined using Steiner and Wits analyses. The Wits analysis was performed to identify cases where the ANB angle (which measures anterior-posterior jaw discrepancies) may not accurately represent malformation due to individual skull and facial structure variations. In cases where the mandible was positioned either anterior or posterior to the cranium or showed clockwise or counterclockwise rotation, the ANB angle could be misleading. If both Wits and Steiner analyses confirmed Class II malocclusion, the patient’s maxillofacial classification was considered Class II. After classifying the maxilla, the occlusal plane and the three ATLs were drawn for each patient. The relationship between the occlusal plane and each of the three ATLs was evaluated by drawing a perpendicular line from each ATL to the occlusal plane. The angles formed between these lines were measured. The line that formed an angle closest to 90 degrees was considered the reference ATL.Statistical analysis was performed to calculate the measured angles’ mean values and standard deviations. The software program SPSS (Version 16.0, Chicago, IL, USA) was used for data analysis. A one-sample t-test was conducted to determine whether the ATLs, ATLm, or ATLi were parallel to the occlusal plane. A significance level of 0.05 was established.

## Results

This study analyzed 172 lateral cephalometric graphs distributed based on jaw classification:

• Class 1: 59 images

• Class 2: 86 images

• Class 3: 27 images

The average angles for the ATLs across the entire sample were:

• Upper tragus line: 85.30°

• Middle tragus line: 87.62°

• Lower tragus line: 89.78°

In the subset of 59 patients with Class 1 jaw classification, a significant difference was observed between the angles formed by the three ATLs, ATLm, and ATLi and the occlusal plane. This variability may influence clinical decisions, such as determining the optimal occlusal plane for dental restorations ( Table 1).

In the 86 patients with Class 2 jaw classification, a significant difference was found between the angle obtained from the three ATLs and the occlusal plane ( Table 2).

In 27 patients with Class 3 jaw classification, a significant difference was observed for the ATLs line (*P* = 0.000), but no significant difference was found for the ATLm and ATLi lines. However, the magnitude of the difference was statistically more significant in ATLi. ( Table 3).

 Table 4 indicates no statistically significant difference between ATLi and the occlusal plane angle (*P* = 0.326) in female patients. However, a significant difference was found between the angles for ATLm and ATLs. Conversely, there was a statistically significant difference for all three ATLs in male patients.in patients aged 15-21, there was no significant difference between ATLi and the occlusal plane. However, a significant occlusal difference was found between ATLm and ATLs. In patients aged 22-45, there was a significant difference between all three ATLs and the occlusal plane.

## Discussion

Determining the location of the occlusal plane in patients requiring complete dentures is not only a challenging task but also a topic of considerable debate. Many practitioners and researchers reference the ATL due to its relative ease of use. However, definitions of the ATL are often confusing because there is no consensus on the exact reference points for this line [7].

This study aims to determine the relationship between the occlusal plane and the ATL as a guide for reconstructing the occlusal plane in patients undergoing complete upper and lower jaw reconstruction across different skeletal classifications (Class 1,2 and 3).This study used lateral cephalometry to explore the relationship between the ATL and the occlusal plane. Lateral cephalometry offers a standardized and accurate method for locating the occlusal plane across all patients [8].

According to the results of this study, in the relationship between the three ATLs and different skeletal classifications, only in Class 3 patients was there statistically significant parallelism between the lower ATL and the occlusal plane. This finding suggests that the inferior tragus line is an accepTable reference for determining the occlusal plane in Class 3 individuals.

However, for Class 1 and Class 2 individuals, none of the three ATLs showed significant parallelism with the occlusal plane, making them unreliable indicators for occlusal plane determination. Notably, the discrepancy with the lower ATL was the smallest in these cases.

Talaeh Ghosn-Abi *et al*. conducted a similar study using cephalometric radiography to investigate the relationship between the three ATLs and the occlusal plane in 20 patients aged 21 to 24, all with Class 1 maxillofacial classification. The results of this study indicated that the middle ATL had the closest relationship with the normal occlusal plane. However, Ghosn-Abi’s study was limited to Class 1 patients, and the sample size was relatively small (20 patients), which may explain the discrepancy in results between their study and the present one [9].

Additionally, Sharab *et al*. [10] conducted a study to evaluate the best reference line among the three ATLs for determining the occlusal plane, considering the effects of age and gender. Their study, involving 58 patients divided into two age groups (22-50 years and 51 years and older), aligned with the findings of our study, concluding that the lower ATL is the most reliable reference for determining parallelism with the occlusal plane. However, Sharab *et al*. did not account for jaw classifications in their study. Furthermore, they observed no significant differences across age groups or between genders, whereas our study found that for women and individuals aged 15-21 years, there was significant parallelism between the inferior tragus line and the occlusal plane. This suggests that the inferior tragus line can serve as a valuable reference for determining the occlusal plane in these groups. One possible explanation for the differences between age ranges in our study could be the effect of age-related tooth loss on occlusal plane disruption.

From the results of this study, it can be concluded that the lower ATL can be used as a reliable reference for determining the occlusal plane, particularly in Class 3 patients. Additionally, age and gender are important factors when using the ATL for occlusal plane determination.

## Conclusions

• Jaw Classifications 1 and 2: Although the parallelism between the lower tragus line and the occlusal plane was not Statistically significant, the findings suggest that this line is relatively closer to the occlusal plane than the other lines.

• Class 3 Classification and General Samples: In Class 3 patients and across all samples, regardless of maxillary classification, the parallelism between the lower tragus line and the occlusal plane was statistically significant. Therefore, the lower tragus line can be a reliable guide for determining the occlusal plane in edentulous patients.

• Gender Differences: In women, there was significant parallelism between the occlusal plane and the lower tragus line, indicating its utility as a guide for determining the occlusal plane in female patients.

• Age Range 15-21: In the 15-21 age group, the parallelism between the occlusal plane and the lower tragus line was significant, supporting its use as a reference for determining the occlusal plane in this age range.

• Combined Gender and Age Factors: In female patients aged 15-21, the lower ATL can confidently guide occlusal plane determination.

## Figures and Tables

**Table 1:** Angles between the Occlusal Plane and the Three ATLs in Cephalometric Skulls (Class I).

Tragus-Ala Line	Mean	Standard Deviation	One-Sample T-Test (P-Value)
ATLs	82.87	3.39	P=0.000
ATLm	84.95	3.39	P=0.000
ATLi	87.45	3.33	P=0.000

**Table 2:** Angles between the Occlusal Plane and the Three ATLs in Cephalometric Skulls (Class II).

Tragus-Ala Line	Mean	Standard Deviation	One-Sample T-Test (P-Value)
ATLs	86.68	3.75	P=0.000
ATLm	89.07	3.79	P=0.026
ATLi	91.26	3.98	P=0.004

**Table 3:** Angles between the Occlusal Plane and the Three ATLs in Cephalometric Skulls (Class III).

Tragus-Ala Line	Mean	Standard Deviation	One-Sample T-Test (P-Value)
ATLs	86.24	4.38	P=0.000
ATLm	88.84	4.36	P=0.181
ATLi	90.17	3.92	P=0.823

**Table 4:** Age-Related Relationship between the Three ATLs and the Occlusal Plane.

Age Group	Tragus-Ala Line	Mean	Standard Deviation	One-Sample T-Test (P-Value)	Sample Size
15-21	ATLs	86.42	4.05	P=0.000	96
	ATLm	88.56	4.08	P=0.001	
	ATLi	90.82	4.12	P=0.052	
22-45	ATLs	83.89	3.78	P=0.000	76
	ATLm	86.43	4.1	P=0.000	
	ATLi	88.47	3.75	P=0.001	

## Data Availability

The datasets used and/or analyzed during the current study are available from the corresponding author.
